# The microbiota-gut-brain axis: a potential target in the small-molecule compounds and gene therapeutic strategies for Parkinson’s disease

**DOI:** 10.1007/s10072-024-07878-x

**Published:** 2024-11-15

**Authors:** Fengjuan Jiao, Lincong Zhou, Zaixin Wu

**Affiliations:** 1 School of Mental Health, Jining Medical University, No. 45, Jianshe South Road, Jining City, Shandong Province 272067 P. R. China; 2 Shandong Collaborative Innovation Center for Diagnosis, Treatment and Behavioral Interventions of Mental Disorders, Institute of Mental Health, Jining Medical University, Jining, Shandong 272067 P. R. China; 3 School of Clinical Medicine, Jining Medical University, Jining, Shandong 272067 PR China

**Keywords:** Parkinson’s disease, Gut microbiota, Neuroinflammation, Immune response, Small-molecule compounds, Therapies

## Abstract

**Backgrounds:**

Parkinson’s disease (PD) is a common neurodegenerative disorder characterized by motor symptoms and non-motor symptoms. It has been found that intestinal issues usually precede motor symptoms. Microorganisms in the gastrointestinal tract can affect central nervous system through the microbiota-gut-brain axis. Accumulating evidence has shown that disturbances in the microbiota-gut-brain axis are linked with PD. Thus, this pathway appears to be a promising therapeutic target for treatment of PD.

**Objectives:**

In this review, we mainly described gut dysbiosis in PD and their underlying mechanisms for mediating neuroinflammation and peripheral immune response in PD pathology and futher discussed the potential small-molecule compounds and genic therapeutic strategies targeting the microbiota-gut-brain axis and their applications in PD.

**Conclusions:**

Studies have found that some small molecule compounds and alterations of inflammation-related genes can improve the motor and non-motor symptoms of PD by improving the microbiota-gut-brain axis, which may provide potentially beneficial drugs and molecular targets for the therapies of PD.

## Introduction

Parkinson’s disease (PD) is the second most common neurodegenerative disease characterized by the motor symptoms bradykinesia, rigidity and tremor, and many non-motor characteristics, accounting for 1% of the world’s population over 60 years of age [1]. The neuropathological features of PD are the loss of dopaminergic neurons and the presence of a-synuclein (α-Syn)-containing Lewy bodies (LBs) in the substantia nigra pars compacta (SNpc) [2]. Generally, genetic factors are thought to be involved in 5–10% of the PD cases [3]. The etiology of PD remains unclear, but is usually due to a combination of genetics, age, and environmental risks [4]. Moreover, there is growing evidence that viral or bacterial exposures, pesticides, and alterations in the gut microbiota play an important role in the pathogenesis of PD [5]. Additionally, it is now understood that patients with PD develop significant neuroinflammation and immune dysfunction at an early age, which is closely associated with a variety of non-motor symptoms such as sleep and gastrointestinal dysfunction [5–7]. The immune dysfunction combined with complex gene-environment interactions may be the major factor that enables the development and progression of PD.

Microbiota can maintain human health by regulating host immunity and promoting intestinal barrier function [8, 9]. Furthermore, growing evidence has shown that the gut microbiota may be the important contributor to central nervous system (CNS) dysfunction [10, 11]. Gut microbiota alterations can increase both intestinal and blood-brain-barrier (BBB) permeabilities, which leads to a continuous accumulation of gut microbiota-derived molecules and metabolites in the brain, thereby promoting neuroinflammation in CNS [12]. Gut dysbiosis has been found to be strongly associated with the pathology of PD in both clinical and preclinical studies [13–16]. The neurotoxin 1-methyl-4-phenyl-1,2,3,6-tetrahydropyridine (MPTP) used for PD animal model can lead to a change in gut microbial composition [14]. Similarly, alterations in the microbiome induced microglial activation and neuroinflammation in mice overexpressing α-Syn, which in turn promote motor dysfunction of the mice [15]. In addition, gastrointestinal dysfunction and gut dysbiosis in PD patients can also interfere with absorption and utilization of many drugs [17–20], suggesting that improving gut dysbiosis in PD may be an important role in treating and preventing the progression of PD. Several therapeutic strategies targeting gut microbiota including fecal microbiota transplantation (FTM), prebiotics and probiotics, and dietary interventions have been validated in animal models and PD patients with the aim of improving symptoms and/or slowing progression of PD [21–28]. It is worth noting that a number of preclinical studies in recent years have identified some small molecule compounds and genetic alterations that can ameliorate the neurodegeneration of dopamine neurons and motor deficits in PD by improving the composition of the gut microbiota and neuroinflammation [29–32], indicating that they may plays protective effects on PD by improving the microbiota-gut-brain axis. In this review, we first introduce the machinery of the microbiota-gut-brain axis. Then, we provide a description of gut dysbiosis in PD and their underlying mechanisms for mediating neuroinflammation and peripheral immune response in PD pathology. Finally, we highlight the potential small-molecule compounds and genic therapeutic strategies targeting the microbiota-gut-brain axis and their applications in PD.

## Gut microbiota and the microbiota-gut-brain axis

Gut microbiota is a complex ecological community that live in the digestive tract of humans and animals, consisting of thousands of microorganisms such as bacteria, viruses and eukaryotes [33]. The composition of the gut microbiota varies in different digestive tracts. There are relatively few bacterial species in the stomach and small digestive tract [34]. However, there is a dense ecosystem of microorganisms containing multiple species in the colon [35]. The colonization of gut microbiota is established at birth, but their composition can be altered by internal and external factors such as diet, antibiotics, bacterial infections, host genetics, and age [36, 37]. Most gut microbiota are beneficial to the human body, including protecting the body from pathogens by production of different antimicrobial substances, enhancing an individual’s immune system, playing an important role in digestion and metabolism [38–40]. Recently, growing evidence has shown that gut microbiota modulates CNS immune responses and functions. Microglia are the tissue macrophages of the brain that are essential for maintaining tissue homeostasis and clearing the pathogens from tissues [41]. Erny et al. reported that global defects in microglia with phenotypic immaturity were observed in germ-free (GF) mice, whereas the microglia impairment can be rectified to the recolonization with a complex microbiota [42]. They also determined that short-chain fatty acids (SCFAs), a bacterial fermentation product, played a critical role in microglia maturation and function [42]. Additionally, it has been found that cyclic SCFAs produced by gut microbiota also enhance the integrity of the BBB by increasing the production of tight junction proteins, further limiting the entry of undesirable substances into the brain [43]. Furthermore, lipoproteins and lipopolysaccharides produced by the gut microbiota can stimulate immune cells to release cytokines, which cross the BBB and activate neurons, leading to changes in mood and behavior of the host [44]. Increased BBB permeability was also observed in GF mice beginning with intrauterine life, which was associated with decreased expression of the tight junction proteins occludin and claudin-5, which regulate barrier function of the endothelial tissue [45]. Taken together, gut microbiota and their metabolites play important roles in maintaining CNS homeostasis and immune responses.

The microbiome-gut-brain axis is a bidirectional communication system between gut microbiota and the nerves system, which can influence brain activity, behavior, development, as well as levels of neurotransmitter receptors through neuroimmune and neuroendocrine mechanisms [46–50]. Gastrointestinal (GI) motility and secretion are regulated primarily by reflexes within enteric nervous system (ENS) in the physiological state, but they are mainly modulated by the autonomic nervous system (ANS) from the brain when the homeostasis of the organism is threatened [51]. The ANS widely innervates most organ systems in the body, which includes the parasympathetic nervous system, the sympathetic nervous system, and the ENS [52]. In the vertebrate gut, the ENS consists of neurons and glial cells arranged in ganglia forming two distinct enteric plexuses and their interconnecting neural pathways [53]. The enteric plexus mainly consists of two neural networks embedded in the intestinal wall: the myenteric plexus located between the longitudinal and circular muscle layers, and the submucosal plexus located in the connective tissues of the submucosa [53]. The ganglionated plexus can provide local neural regulation on tissues and cells adjacent to the ganglia. Since ENS neurons do not extend into the intestinal lumen, they sense the gut microbiota primarily through microbial molecules that penetrate the epithelial barrier and the epithelial cells themselves [54]. Certain microbial components, microbiota-regulated hormones, and microbiota-dependent immune mediators can directly interact with the enteric neurons as well as vagal and spinal afferents innervating the gut. The local signals can be transmitted through sensory neural circuits to brain regions involved in cognition, emotion, fear/anxiety, somatic sensations, and/or feeding behavior [55–57]. In turn, the vagal and spinal efferents nerves transmit signals to the intestinal mucosa and affect gastrointestinal homeostasis through indirect interactions with the ENS, ultimately influencing local immune function and the composition of the gut microbiota [58].

In addition to ENS sensing of microbial molecules, enteroendocrine cells (EECs) and enterochromaffin cells (ECCs) also form an important intermediate in microbiota-ENS signaling. Microbial products and metabolites (e.g., secondary bile acids, indole derivatives, and SCFAs) can signal through EECs and ECCs to regulate the secretion of several neuropeptides and neuromodulators such as the appetite-regulating hormone GLP1, hormones, and neurotransmitters. If these secretions cross the BBB, they can directly affect the activity of central neurons [59]. Furthermore, it has been reported that the gut microbiota has bidirectional interactions with the neuroendocrine signaling pathway mediated by the hypothalamic-pituitary-adrenal (HPA) axis [60]. Stress factors can increase the release of corticotropin-releasing hormone (CRH) from the hypothalamus, which subsequently stimulates the secretion of adrenocorticotropic hormone (ACTH) from the anterior pituitary gland. Under the influence of ACTH, the adrenal glands begin to secrete cortisol, which can impact the microbiota-gut-brain axis through multiple pathways [61]. Studies have found that cortisol receptors are expressed in a variety of intestinal cells, including epithelial cells, EECs, and immune cells, which suggest that cortisol may have direct effects on intestinal function [62–65]. In addition, cortisol can also change the composition and diversity of the gut microbial genome by altering intestinal transit time, intestinal permeability, and nutrient supply [65]. Conversely, gut dysbiosis can also alter HPA axis function. Mechanistically, gut dysbiosis may lead to increased release of cytokines including interleukin (IL)-1β, IL-6, and tumor necrosis factor-α (TNF-α), which may cross the BBB and activate the HPA axis [66, 67]. Lipopolysaccharide (LPS) and peptidoglycan (a component of the cell wall of most bacteria) released by gut microbes can also activate the HPA axis [68, 69]. Abnormalities in the control or integration of interoceptive signals can lead to perturbations throughout the entire microbiota-gut-brain axis system [70]. A growing body of studies have shown that abnormalities in the microbiota-gut-brain axis are associated with intestinal disorders such as irritable bowel syndrome (IBS), functional dyspepsia, chronic abdominal pain, as well as psychiatric and neurodegenerative disorders [71–76].

## Gut microbiota and inflammation in PD pathology

### Gut dysbiosis in PD

Gut dysbiosis, which is defined as alterations in the structure and/or function of the gut microbiota, have been reported in patients diagnosed with neurological disorders [62, 77]. GI dysfunction, especially constipation, is an important nonmotor symptom of PD that usually precedes the motor symptoms by several years [78]. Many studies have confirmed the correlation between GI dysfunction of PD and composition of gut microbiota, which have discovered that there was a diversity of microbiota in the feces of patients with PD [79–82] (Table 1). Results from a clinical study showed the abundance of *Prevotellaceae* in the feces of patients with PD reduced by 77.6% compared to healthy controls, which also was significantly correlated with the Unified Parkinson’s Disease Rating Scale (UPDRS)-III total score [83]. Furthermore, they also found that the relative abundance of *Enterobacteriaceae* were significantly increase in patients with a postural instability and gait difficulty (PIGD) phenotype and was positively associated with the severity of postural instability and gait disturbance [83]. Gut microbial analysis of 89 PD patients in Siberia showed changes in the content of 9 genera and 15 species of microorganisms in the intestines of patients compared to healthy controls. There was a decrease in *Dorea*, *Bacteroides*, *Prevotella*, and *Faecalibacterium* and an increase in *Christensenella*, *Catabacter* and *Lactobacillus*. This gut microbial pattern may trigger the localized inflammation, which in turn leads to aggregation of α-Syn and the production of LBs [84]. A study by Liu and his colleagues has shown a significant difference in the fecal microbiota between tremor and non-tremor subtypes in 80 PD patients in Taiwan, China. The relative abundance of *Clostridium*, *Verrucomicrobia*, and *Akkermansia* in the feces of PD patients with the tremor subtype was higher than that of the non-tremor subtype, whereas *Propionibacterium*, *Bacteroidia*, *Flavobacterium*, *Mogibacterium*, *Sutterella*, *Alcaligenacea Cupriavidus*, and *Desulfovibrio* were more abundant in the non-tremor subtypes. Moreover, *Lactobacillus* abundance was correlated with the severity of motor symptoms in patients [85]. In another clinical study included 24 patients with PD and 14 healthy volunteers, it was reported that putative cellulose degraders from the genera *Blautia*, *Faecalibacterium* and *Ruminococcus* were reduced in the feces of patients with PD, while putative pathobionts from the genera *Escherichia-Shigella*, *Streptococcus*, *Proteus*, and *Enterococcus* were increased, which may lead to decreased production of SCFAs and increased production of endotoxins and neurotoxins [86]. A meta-analysis of 10 case-control studies with a total of 1,703 subjects showed that the composition of the microbiome of PD patients was significantly different from that of controls, including an increase in the rare taxa *Megasphaera* and *Akkermansia* and a decrease in the abundant of *Roseburia* genus [87]. They also found that many bacterial genera are associated with PD motor severity and cognitive function [87]. Weis et al. observed that several bacterial taxa linked to health-promoting, anti-inflammatory and neuroprotective, such as *Faecalibacterium* and *Fusicatenibacter*, reduced in 34 PD patients [88]. Additionally, the *Clostridiales family XI* and their affiliated members which were capable of peptone and amino acid fermentation, including *Peptoniphilus* and *Finegoldia*, also increased in PD patients [88–90]. However, they didn’t find any difference of the beta diversity between PD and control samples. By analyzing the shotgun metagenome sequencing data obtained from the Sequence Read Archive (SRA) database, Yu et al. found significant differences in the diversity, abundance and composition of the gut microbiota of PD patients compared to healthy individuals [91]. Notably, their study in a 6-hydroxydopamine (6-OHDA)-induced rat model of PD also observed that gut microbiota dysbiosis exacerbated behavioral deficits and oxidative stress through inhibition of the expression of nicotinamide mononucleotide adenylyl transferase 2 (NMNAT2), a key enzyme that catalyzes the synthesis of nicotinamide adenine dinucleotide (NAD^+^) from NMN [91, 92]. Previous studies have reported that α-Syn pathologically overexpressed in intestinal tissues of PD mouse model [93]. Thus, we speculated that the overexpression of α-Syn in the gut may be responsible for the increase in these genera, but further studies are needed to explore the metabolic activity of these genera with respect to α-Syn and their interaction with ENS. Additionally, the administration of drugs such as levodopa and entacapone to PD patients also significantly affects the relative abundance of the genera *Peptoniphilus*, *Finegoldia*, *Faecalibacterium Fusicatenibacter*, *Anaerococcus*, *Bifidobacterium*, *Enterococcus* and *Ruminococcus*. Whether these alterations in bacterial taxa affect the metabolism and absorption of dugs further needs to be investigated [88]. A study has also reported that significantly high levels of *Rikenellaceae* and *Turicibacteraceae* were found in the gut microbiota of 59 patients suffering from PD for > 1 year and 13 new PD patients compared to the corresponding healthy controls, respectively. Moreover, the genera *Turicibacter* and *Prevotella* were correlated with the PD severity [94]. Baldini et al. discovered that PD-associated microbial patterns were associated with gender, age, body mass index, and constipation in PD patients. And the relative abundances of *Bilophila* and *Paraprevotella* were significantly associated with the Hoehn and Yahr staging [95]. *Desulfovibrio* bacteria (DSV) belongs to sulfate-reducing bacteria (SRB) which can cause infections in humans. Hydrogen sulfide (H2S) produced by DSV can induce α-Syn aggregate formations [96, 97]. Results from the detection of faces from 20 PD patients, Murros et al. found that all PD patients had higher levels of *Desulfovibrio* in their gut microbiota than healthy controls. And the concentration of *Desulfovibrio* was significantly correlated with the severity of PD [98]. In a Chinese case-control study with 45 PD patients, genera *Clostridium IV* and *XVIII*, *Butyricoccus*, *Aquabacterium*, *Holdemania*, *Sphingomonas* and *Anaerotruncus* were enriched in the PD patients gut compared to healthy controls. Among those flora, genera *Butyricicoccus* and *Clostridium XlVb* are related to cognitive impairment in PD patients. Furthermore, genera *Escherichia/Shigella* were negatively associated with PD duration [82]. Taken together, those studies indicate that gut dysbiosis is closely related to PD clinical symptoms, and altering the gut microbiota may be a potentially therapeutic option of PD. However, the relationship between the relative abundance of the microbiota and disease progression in patients with PD is not consistent, possibly due to differences in methodology, race, region, age, and other factors. For example, local diets vary between different countries and regions, and different diets can lead to significantly different compositions of the gut microbiota [99, 100].

**Table 1:** The clinical correlations between altered gut microbiota and PD

Type of study	Study subjects	Gene amplification	Altered microbiota	Clinical correlations	Ref.
A case-control study	72 PD patients and 72 controls	The V1-V3 regions of the bacterial 16 S ribosomal RNA gene	*Prevotellaceae*↓; *Enterobacteriaceae↑;*	The severity of postural instability and gait disturbance	[83]
A cross-sectional cohort study	197 PD patients and 103 controls	The bacterial 16 S rDNA V4 region	*Christensenellaceae*↑; *Desulfovibrionaceae*↑; *Bifidobacterium*↑; *Collinsella*↑; *Bilophila*↑; *Akkermansia*↑; *Lachnospiraceae*↓; *Roseburia*↓; *Lachnospiraceae*↓; *Faecalibacterium*↓	Firmer stool consistency and constipation severity	[79]
A prospective observational case-control study	193 idiopathic PD patients, 22 progressive supranuclear palsy (PSP), 22 multiple system atrophy (MSA**)**, and 103 controls	The V3-V4 region of the 16 S ribosomal RNA gene	*Lachnospiraceae*↓;*Lactobacillaceae*↑↓ *Christensenellaceae*↑	Cognitive impairment; Gait; Disturbances; and postural instability	[80]
A case-control study	51 PD patients and 48 controls	The V4 region of the 16 S ribosomal RNA gene	*Akkermansia↑;Prevotella↑↓* *Prevotella copri↑;Lactobacillales/Lactobacillaceae/Lactobacill↓↓* *Lactobacillus↓*	Clinical scores, such as UPDRS, NMSQ and SCOPA	[81]
A case-control study	45 patients and 45 healthy spouses	The V3-V4 region of 16 S ribosomal RNA gene	*Clostridium IV↑; Aquabacterium↑; Holdemania↑; Sphingomonas↑; Clostridium XVIII↑; Butyricicoccus↑; Anaerotruncus↑;Lactobacillus↓;* *Sediminibacterium↓*	Disease duration; levodopa equivalent doses (LED)↓Cognitive impairment	[82]
A case-control study	24 PD patients and 14 healthy volunteers	The V3-V5 region of 16 S ribosomal RNA gene	*Blautia↓; Faecalibacterium↓;* *Ruminococcus↓;* *Escherichia-Shigella↑;* *Streptococcus↑; Proteus↑; Enterococcus↑*	Disease severity and PD duration	[86]
A case-control study	89 patients and 66 patients without severe somatic pathology and manifestations of parkinsonism (control group)	The V3-V4 region of 16 S ribosomal RNA gene	*Dorea↓; Bacteroides↓; Prevotella↓; Faecalibacterium↓; Bacteroides massiliensis↓; Stoquefichus massiliensis↓; Bacteroides coprocola↓; Blautia glucerasea↓; Dorea longicatena↓; Bacteroides dorei↓; Bacteroides plebeus↓; Prevotella copri↓; Coprococcus eutactus↓; Ruminococcus callidus↓;* *Christensenella↑; Catabacter↑; Lactobacillus↑; Oscillospira↑; Bifidobacterium↑; Christensenella minuta↑; Catabacter hongkongensis↑; Lactobacillus mucosae↑; Ruminococcus bromii↑; Papillibacter cinnamivorans↑*	Aggregation of α-synuclein and generation of Lewy bodies	[84]
A case-control study	80 PD patients and 77 controls	The V3-V4 region of the 16 S ribosomal RNA gene	*Bacteroide↑;* *Prevotella↓;* *Parabacteroides↑*,* Verrucomicrobia↑*,* Akkermansia↑*,* Butyricimonas↑*,* Veillonella↑*,* Odoribacter↑*,* Mucispirillum↑*,* Bilophila↑*,* Enterococcus↑*,* and Lactobacillus↑*	Tremor; Motor symptom; Severity	[85]
A case-control study	34 PD patients and 25 controls	The V4 and V5 hypervariable region of bacterial 16 S ribosomal RNA gene	*Faecalibacterium↓;* *Fusicatenibacter↓;* *Clostridiales family XI↑*	Medication with L-dopa and entacapone	[88]
A case-control study	59 patients suffering from PD for > 1 year (OPD), 13 new PD (NPD) patients, 68 controls	The 16 S ribosomal RNA gene	*Rikenellaceae↑;* *Turicibacteraceae↑;* *Butyricimonas↑; Parabacteroides↑; Christensenellaceae R-7 group↑; Ruminococcaceae UCG↑; Alistipes↑*	PD duration and disease severity	[94]
A case-control study	147 PD patients and 162 controls	The V3-V4 regions of the 16 S ribosomal RNA gene	*Akkermansia muciniphila↑; Lactobacillus↑;* *Turicibacter↓*	Host metabolism and disease phenotype	[95]

In addition, gut dysbiosis has also been found in animal models of PD. For example, in a mouse model of PD treated with rotenone, 16 S rRNA sequencing of fecal microbiome showed an overall decrease in bacterial diversity and significant changes in microbial composition. Moreover, the changes of fecal microbiota composition induced by rotenone were strongly associated with gastrointestinal and motor dysfunction in mice [101]. Likewise, gut microbial alpha diversity was altered in rotenone-treated Sprague-Dawley rats. The genera *Actinobacteria* and *Proteobacteria* were increased, while genera *Bacteroidetes*, *Cyanobacteria* and *Firmicutes* were decreased in rotenone-treated rats, which is relatively consistent with alternations reported in patients with PD [102]. Gut microbiota could also promote α-Syn pathology, motor and GI dysfunction of the thy1-aSyn-overexpressing (ASO) mouse [15]. Additionally, transplanting the gut microbiota derived from PD patients into ASO mouse further aggravated the motor dysfunction. This result further indicates that changes in the human microbiome may be a risk factor for PD duration. Recently, a study showed that the abundance of *Escherichia coli (E. coli)* was significantly increased in LRRK2 R1628P and G2385R mice. The LRRK2 variant (R1628P) increased phosphorylation of α-Syn caused by curli in *E. coli*-derived extracellular vesicles. Moreover, *E. coli* administration triggered pathologic α-Syn aggregation in the colon and diffusion to the brain via the microbiota-gut-brain axis in LRRK2 mutant mice [103].

### Gut microbiota and inflammation in PD

**Fig. 1:**
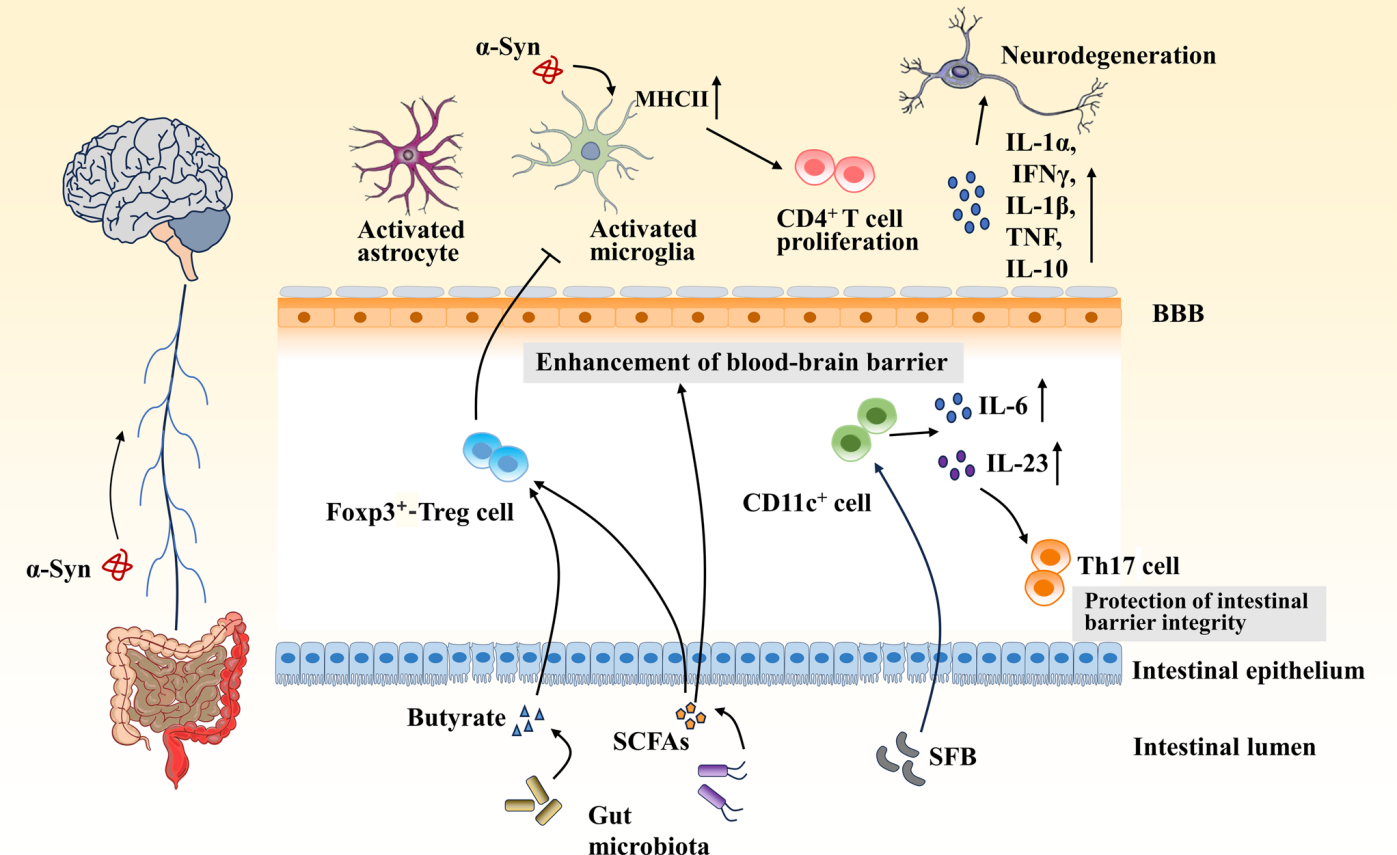
A potential inflammatory mechanism of gut microbiota in PD. Gut dysbiosis in PD can lead to accumulation of α-Syn in the gut, which can enter the brain via the vagus nerve. Gut microbiota and their metabolites modulate intestinal inflammation and activate astrocytes and microglia, which in turn release pro-inflammatory factors leading to dopaminergic neuronal damage. Cyclic SCFAs produced by gut microbiota can enhance the integrity of the BBB. Furthermore, SCFAs and butyrate may inhibit intestinal inflammation and neuroinflammation by inducing FoxP3^+^- Treg cell differentiation. Adhesion of the symbiotic mucosa-associated segmented filamentous bacterium (SFB) ‘Candidatus Arthromitus’ to intestinal epithelial cells acts on CD11c^ +^ cells in the lamina propria to stimulate the production and release of interleukin (IL)-6 and IL-23, and thereby stimulate the differentiation and activation of Th17 cells, which play an important role in the protection of intestinal barrier integrity and the maintenance of intestinal homeostasis

The research community proposes that the development of PD is accompanied by the activation of the innate immune and adaptive immune systems, which changes dynamically with disease progression, contributing to neuronal degeneration in the brain [5]. Growing evidence suggests that gut dysbiosis plays a key role in the development of neuroinflammatory and immune responses in PD (Fig. 1).

#### Gut microbiota, CNS inflammation and PD

Microglia, tissue-resident macrophages in the brain, are densely distributed in the SNpc and striatum of the brain involved in PD. It has been showed that overactivation of microglia can lead to CNS inflammation and dopamine neuron damage [104]. McGeer et al. first found human leukocyte antigen (HLA)-DR^+^ (a major histocompatibility complex class II (MHC-II) cell surface receptor) responsive microglia in postmortem tissues of PD patients [105], and the number of the HLA-DR^+^ microglia increased as the neuronal degeneration of the SNpc proceeded [106]. In addition, α-Syn can activate microglia through the Toll-like receptor 4 (TLR4) or the TLR2 pathway and promote the production of pro-inflammatory factors such as nitric oxide (NO), and TNF and interferons(IF), which in turn produce toxic effects on dopaminergic neurons [107–109]. Similarly, studies of patients with PD have also found elevated levels of the proinflammatory cytokines related to the risk of PD in the brain tissue and cerebrospinal fluid (CSF), including TNFα, transforming growth factor (TGF)-β1, IL-6, and IL-1β [85, 110, 111]. These findings suggest that microglia overactivation plays an important role in neuroinflammation and neurodegeneration in PD.

Growing studies have suggested that the inflammation leading to neurodegeneration may be related to gut dysbiosis [60, 112, 113]. Erny et al. found that a diverse gut microbiota is necessary to maintain microglia maturation, morphology and immune function. The absence of a host microbiota can lead to defects in microglia maturation, activation and differentiation as well as compromised immune response to bacterial or viral infections [42]. Within caudoputamen (CP) and substantia nigra (SN), microglia in germ-free wild type (GF-WT) mice displays a maturation arrest and/or a reduced activation state, suggesting that gut microbiota affect immune cells in the brain [15]. Furthermore, antibiotic-treated specific pathogen-free-alpha-synuclein-overexpressing (SPF-ASO) mice display higher maturation of microglia and significantly increased levels of the pro-inflammatory cytokines TNF-α and IL-6 compared with germ-free-α-Syn-overexpressing (GF-ASO) mice, indicating that gut microbiota can promote α-Syn-dependent activation of microglia [15]. In MPTP-induced PD mouse models, sodium butyrate, a short-chain fatty acid, was found to attenuate PD-associated BBB disruption by upregulation of occludin and zonula occludens (ZO)-1 [114]. In addition, an in vitro data showed that SCFAs protected the integrity of the BBB through direct effects on endothelial cells and activation of anti-inflammatory pathways [115]. Huuskonen et al. found that butyrate could induce an adaptative response against microglial activation [116]. Interestingly, butyrate performed a significant protective effect on LPS-induced inflammatory response in rat primary microglia. However, in the transformed N9 microglial cell line, sodium butyrate enhanced the LPS-induced inflammatory response and downregulateed the DNA binding capacity of NF-κB transcription factor induced by LPS stimulation [116]. It has been observed that SCFAs could promote the activation of microglial cells and leaded to enhanced motor deficits in GF mice overexpressed α-Syn [15]. Interestingly, a significant reduction in the number of SCFA-producing bacteria and fecal excretion of SCFA has also been detected in fecal samples from PD patients and animal models, which may be an important mechanism for the abnormal neuroinflammation in PD [13, 117].

The above studies have shown that microbial products and metabolites could promote the maturation and activation of microglia, indicating that gut dysbiosis in PD patients may be an important mechanism for causing excessive CNS inflammation. Alteration of the microbial composition of PD patients through diet or medication may be a potential method to improve the DAergic neurodegeneration induced by the abnormal inflammation.

#### Gut microbiota, peripheral inflammation and PD

Dysregulation of both cellular immune responses and humoral immune responses in the periphery has been observed in PD patients and animal models [118, 119]. Data from several lines of preclinical and clinical studies has shown that the pathological process of PD is associated with alterations in the number and function of peripheral immune cell populations. A study of samples from 41 patients with PD showed that the phagocytic capacity of monocytes in the peripheral blood of patients with early-moderate PD was increased compared with controls [120]. In a mouse model induced by overexpression of α-Syn, Harms et al. observed that α-Syn induced microglia activation, antigen presentation, IgG deposition and dopaminergic neuronal degeneration by upregulating MHCII expression in microglia. In the in vitro systems, they also found that aggregated α-Syn activated the antigen processing and antigen presentation capacity of microglia, which in turn drove CD4 T cell proliferation and triggers the release of cytokines such as IL-1α, IFNγ, IL-1β, TNF and IL-10 [121]. In addition, they also found that α-Syn induced a robust infiltration of CCR2^+^ peripheral monocytes into the SN, whereas deletion of CCR2 can prevent α-Syn-induced monocyte entry, attenuate MHCII expression, and decrease degeneration of dopaminergic neurons, suggesting that extravasation of pro-inflammatory peripheral monocytes into the CNS play an important role in neuroinflammation and neurodegeneration in PD [122]. There is an increase in effector/memory T cells (Tem) and an impaired abilities of regulatory T cells (Treg) to suppress effector T cell function in the peripheral blood of patients with PD, which linked to PD pathobiology and disease severity [123]. A study in 2018 reported that the number of T lymphocytes increased in postmortem PD brain tissues. Furthermore, activated T lymphocytes producing IL-17 were found to promote neuronal death in autologous co-cultures of activated T lymphocytes and iPSC-derived midbrain neurons of sporadic PD patients [124]. In addition, increased numbers of CD3^+^/CD4^+^ T cells near microglia and astrocytes were detected in the brains of α-Syn transgenic models, implying that these infiltrating peripheral adaptive immune cells are involved in the process of activating immune cells in the CNS to enhance the neuroinflammatory response [125]. T helper (Th)17 cells are a subset of CD4^+^ T lymphocytes with strong proinflammatory property [126]. In MPTP-induced PD mouse models, Th17 cells were found to invade into SNpc where BBB was disrupted [127]. In addition, Th17 cells directly exacerbated DAergic neuronal loss through LFA-1/ICAM-1 interaction in MPP^+^-treated ventral mesencephalic (VM) cell cultures [127]. CD4^+^CD25^+^ Tregs are a subpopulation of CD4^+^ T cells that specifically express the transcription factor FoxP3 in the nucleus and CD25 and CTLA-4 on the cell surface, and play essential functions in suppressing immune activation and maintaining immune homeostasis and tolerance [128]. CD3-activated Tregs were demonstrated to protect against dopaminergic neuronal loss through inhibition of microglial oxidative stress and inflammation induced by activated microglia in a MPTP mouse model of PD [129].

Recently, there is growing supports for the idea that intestinal inflammatory processes and gut-derived inflammation associated with dysbiosis play a pathogenic role in PD [130, 131]. Several lines of evidence have shown that abnormally altered gut microbiota in PD contributed to neuroinflammation and neurodegeneration by affecting the differentiation and proliferation of T cells in the gut. *Faecalibacterium* and *Roseburia*, which are reduced in PD, produce butyrate, which exerts potent effects on many colonic mucosal functions such as inhibiting inflammation, reinforcing the defense function of the colon and decreasing oxidative stress [132, 133]. Butyrate enhances the acetylation of Foxp3 protein and reduces the expression of pro-inflammatory factors in dendritic cells (DCs) through inhibition of histone deacetylase (HDAC), thereby promoting Treg production [134, 135]. Similarly, propionate, another SCFA of microbial origin capable of HDAC inhibition, also promotes de novo Treg-cell generation in the periphery in vivo [134]. In addition, it has been reported that SCFAs also can increase colonic Treg population size and function and protect against colitis in a Ffar2(GPR43)-dependent manner [136]. In colon, butyrate induces the differentiation of Treg cells and IL-10-producing CD4^+^ T cells through acting on the G protein-coupled receptor GPR109a which expressed on dendritic cells and macrophages [137]. Furthermore, butyrate can interact on the GPR109a receptor in colonic epithelial cells and induce the production of IL-18, and subsequently suppresses colonic inflammation [137]. Thus, a decrease in the abundance of butyrate-producing bacteria in PD may exacerbate neurodegeneration by failing to suppress neuroinflammation. In the mice colonized with the symbiotic mucosa-associated segmented filamentous bacterium (SFB) ‘*Candidatus Arthromitus*’, Omenetti et al. observed that adhesion of SFB to intestinal epithelial cells acted on CD11c^+^ cells in the lamina propria to stimulate the production and release of IL-6 and IL-23, and thereby stimulated the differentiation and activation of Th17 cells, which played an important role in the protection of intestinal barrier integrity and the maintenance of intestinal homeostasis [138, 139]. In the terminal ileum, commensal Th17 cells induced by SFB exerted unique anti-inflammatory effects and regulated effector T cell responses through expression of transcription factor c-MAF and the cytokine IL-10 [140]. Beta-N-methylamino-L-alanine (BMAA), a natural proteinogenic diamino acid produced by cyanobacteria, diatoms and methanogens, is usually detected in its free form [141]. Although there is no evidence that human gut microbiota can produce BMAA, a study has reported that hypermethylation in the promoter region of the SLC7A11 gene in patients with PD is link to downregulation of the cysteine-glutamate antiporter, a target of BMAA, which is thought to be consistent with environmental risks related to PD [142]. A recent study observed that mice were orally administered with BMAA for 12 weeks significantly reduced the abundance of microbiota SFB in the ileal mucosa, leading to increased intestinal inflammation and loss of intestinal barrier integrity. Surprisingly, BMAA treatment induced propagation of α-Syn aggregates from the gut to the SN region of the brain via the vagus nerve, which in turn triggered neuroinflammation, dopaminergic neuron degeneration, and movement disorders [143]. This leads to the conclusion that the regulation of Th17 by SFB may play a fundamental role in the progression of generation of α-Syn aggregates and the occurrence of neuroinflammation, but further investigations are needed by more studies. Recently, it has been found that *Subgingival plaque* (LIP SP) caused an increased abundance of *Veillonella parvula* (*V. parvula*) and *Streptococcus mutans* (*S. mutans*) in the feces of MPTP-induced PD mice, leading to activation of microglia in the brain, and proliferation of T helper 1 (Th1) cells in the brain and gut. In *V.parvula* - and *S.mutans* -treated PD mice, administration of IFNγ protected dopaminergic neurons from damage caused by dysbiosis [144].

## Therapies targeting the gut microbiota in PD

Several lines of evidence have identified gut microbiota disturbances in patients with PD, which can also affect levodopa absorption [20]. In addition, several therapeutic strategies targeting gut microbiota have been validated in preclinical animal models and PD patients with the aim of improving symptoms and/or slowing progression of PD. In recent years several evidence have found that many drugs, natural small molecule compounds and deletion of inflammation-related genes also exert anti-inflammatory and neuroprotective effects by modulating the gut microbiota (Table 2).

**Table 2:** Effects of drugs and small molecule components on gut microbiota in PD

Compounds/Drugs	Methods of administration and dosage		Animal models	Altered microbiota	Effects	Ref.
**Clinical drugs**						
Ceftriaxone	Intraperitoneal injection, 200 mg/kg		MPTP-treated mice	*Proteus*↓ *Akkermansia*↑	Alleviate MPTP-induced activation of astroglia and microglia; Reduce expression of TLR4, MyD88, p-NF-κB in the brain and colon; Decrease the serum concentration of IL-1β, IL-6, and TNF-α	[146]
Cinnabar and Realgar	Oral, HFD-original: 0.06 g/kg/day, HFD-reduced: 0.018 g/kg/day		Lipopolysaccharide (LPS) plus rotenone (ROT)-induced rat model	*Verrucomicrobiaceae*↓ *Lactobacteriaceae*↓ *Enterobacteeriaceae*↑	Rescue LPS- and ROT-induced DA neurons loss; Improve motor dysfunction of the rats; Attenuate activation of microglia	[149]
***Natural small molecule compounds***						
Dihuang Granule	Oral, 10 g/kg/day		MPTP-treated mice	*Proteobacteria*↓ *Patescibacteria*↓ *Muribaculum*↑ *Turicibacter*↑ *Lactobacillus*↑ *Ruminococcaceae*↑ *Candidatus_Saccharimonas↑* *Enterorhabdus*↑	Improve the damage of dopaminergic neurons; Ameliorate motor impairments; Suppress PD-associated inflammation and oxidative stress	[31]
Curcumin	Oral, 100 mg/kg/day		MPTP-treated mice	*Muribaculaceae*↑ *Lactobacillaceae*↑ *Lachnospiraceae*↑ *Eggerthellaceae*↑ *Aerococcaceae*↓ *Staphylococcaceae*↓	Improve motor deficits, glial cell activation, and the aggregation of α-syn; Upregulate the levels of tyrosine, methionine, sarcosine and creatine	[32]
Diosgenin	Oral, 80 mg/kg/day		MPTP-treated mice	*Firmicutes-to-Bacteroidetes ratio*↓ *Enterococcus*↓ *Streptococcus*↓ *Bacteroides*↓ *Lactobacillus genera*↓	Improve motor behavior; Inhibit neuron viability and oxidative stress; Promote bile acid (BA) -mediated GLP-1 pathway	[167]
Resveratrol	Oral, 30 mg/kg/day		MPTP and probenecid (MPTP/P) -treated mice	*Prevotellaceae*↑ *Rikenellaceae*↑ *Erysipelotrichaceae*↑ *Fimicutes-to-Bacteroidetes ratio*↓ *Lachnospiraceae*↓ *Akkermansia*↓	Improve MPTP/P-induced behavioral performance; Prevent MPTP/P-induced dopaminergic neurodegeneration	[174]

### Drugs

#### Ceftriaxone

Ceftriaxone is a new third-generation cephalosporin commonly used in clinical practice, which has excellent activity against many Gram-negative and Gram-positive microorganisms [145]. Recently, a study showed that intraperitoneal injection of ceftriaxone (200 mg/kg) for 7 days alleviated MPTP-induced activation of astroglia and microglia in the substantia nigra, reduced the expression of neuroinflammation-related TLR4, myeloid differentiation primary response 88 (MyD88), and phosphorylated NF-κB (p-NF-κB) in the brain and colon of PD mice, and decreased the serum concentration of IL-1β, IL-6, and TNF-α [146]. They further demonstrated that ceftriaxone reduced the abundance of the *Proteus* genus and increased the abundance of *Akkermansia*, which suggests that ceftriaxone may exert neuroprotective effects by regulating inflammation and gut microbiota [146].

#### Hua-Feng-Dan (HFD)

Hua-Feng-Dan (HFD) is a traditional Chinese medicine containing a variety of components, which has been used to treat stroke and PD [147]. Previous studies have indicated that cinnabar and realgar were the main active components of HFD in in vitro [147, 148]. In a chronic LPS plus rotenone (ROT)-induced rat model, Chen et al. found that the HFD-original (containing 10% cinnabar and 10% realgar, 0.06 g/kg/d) rescued LPS-and ROT-induced loss of DA neurons, improved motor dysfunction of the rats and attenuated the activation of microglia in the substantia nigra tissue [149]. In addition, the results from gut microbiome analysis demonstrated that HFD-original ameliorated the LPS- and ROT-induced the increased abundance of *Verrucomicrobiaceae* and *Lactobacteriaceae* genera and the decreased abundance of *Enterobacteeriaceae* genera [149]. Thus, the active ingredients cinnabar and realgar in HFD may have a protective effect on neuronal degeneration and improve the composition of gut microbiota in PD models.

### Natural small molecule compounds

#### Dihuang Granule

Compound Dihuang Granule (CDG) is another traditional Chinese medicine used in the treatment of PD. Several preclinical evidence has shown that CDG can effectively improve neurotoxin-induced upregulation of inflammatory factors, neuronal degeneration, motor dysfunction and GI dysfunction [150, 151]. Recently, it has been reported that orally administrated 10 g/kg/day CDG in PD mouse models can ameliorate MPTP-induced gut microbial dysbiosis, inflammatory responses in the CNS and colon via blocking the TLR4/NF-κB pathway, which in turn protected the intestinal barrier responses [31].

#### Curcumin

Curcumin (CUR) is a low molecular weight polyphenol compound derived from turmeric that has been found to have anti-inflammatory, antioxidant and anticancer properties [152]. It was previously found that CUR exerts neuroprotective effects in PD through inhibition of reactive oxygen species (ROS) production, microglial activation, and production of α-Syn aggregation [153–155]. In addition, CUR can play a protective role in many diseases by restoring microecological dysregulation and improving intestinal barrier function [156, 157]. Cui et al. found that given through intragastric administration of CUR (100 mg/kg/day) daily for 4 weeks effectively improved MPTP-induced motor deficits, glial activation, and the aggregation of α-Syn. The results from 16 S rRNA sequencing showed that the abundance of *Muribaculaceae*, *Lactobacillaceae*, *Lachnospiraceae*, and *Eggerthellaceae* was increased, while the abundance of *Aerococcaceae* and *Staphylococcaceae* was decreased in CUR-treated mice compared with MPTP mice. Furthermore, serum metabolomics analysis showed that CUR treatment resulted in a rapid increase in tyrosine and levodopa (dopa) levels in the brain, and that these changes were associated with the abundance of *Lactobacillaceae* and *Aerobacteriaceae*, suggesting that CUR can ameliorate the progression of PD by modulating the gut microbiota-metabolite axis [32].

#### Diosgenin

Diosgenin, a natural steroidal saponin found primarily in *Dioscorea* species, has been shown to have strong anti-inflammatory and antioxidant activity [158, 159]. Growing evidence has shown that diosgenin show great potential in neuroprotection and regulation of gut microbiota [160, 161]. For example, it can attenuate amyloid-β (1–42)-induced neurodegeneration [162], ameliorate LPS-induced microglial activation and neuronal damage [163], and ameliorate murine colitis by regulating macrophage polarization and recovering the disturbed gut microbiota [164, 165]. Interestingly, in a study on melanoma, the anticancer effects of diosgenin were more dependent on inducing antitumor immunity by modulating gut microbiota composition [161]. In a mouse model of pentylenetetrazole (PTZ)-induced epilepsy, diosgenin (80 mg/kg/day) treatment reversed PTZ-induced decrease in the abundance of *Bacteroides* and *Parabacteroides* genera, inhibited the activation of enteric glial cells (EGCs) and the TLR4-MyD88 pathway, which in turn reduced the expression of pro-inflammatory factors in the colon and improved the intestinal barrier function, and ultimately inhibited the progression of epilepsy [166]. A recent study found that administered intragastrically with diosgenin (80 mg/kg/day) restored MPTP-induced gut dysbiosis to decrease *Firmicutes*-to-*Bacteroidetes* ratio and the abundances of *Enterococcus*, *Streptococcus*, *Bacteroides* and *Lactobacillus* genera by upregulating the GLP-1 signaling pathway, which further inhibited bile acid-mediated oxidative stress and neuroinflammation, significantly improving the pathological phenotype of PD mice [167].

#### Resveratrol

Resveratrol, a polyphenol compound found in many plant species, has a variety of biological properties including anti-inflammatory, antioxidant, anticancer and neuroprotective properties [168]. Experimental studies in in vitro and in vivo have shown the neuroprotective effects of resveratrol on PD [169]. Many studies of the pharmacological function of resveratrol found that most of resveratrol could not be absorbed in the small intestine, but it could interact with the gut microbiota, regulate the composition of the gut microbiota, and undergo biotransformation to active metabolites via the gut microbiota [170–173]. FMT from resveratrol-treated (30 mg/kg/day) PD mice to MPTP-induced PD mouse models showed that FMT could increase the abundance of *Prevotellaceae*, *Rikenellaceae*, and *Erysipelotrichaceae* genera, decreased the ratios of *Fimicutes/Bacteroidetes* and the abundance of *Lachnospiraceae* and *Akkermansia* genera, which may contribute to the neuroprotective effects in PD through increasing the number of TH-positive neurons in the SNpc and enriched TH-positive fiber density in the striatum [174].

### TLR2 and TLR4

Toll-like receptors (TLRs), a family of pattern recognition receptors (PRRs), are observed in glial cells and neurons that respond to invading exogenous pathogens and endogenous pathogens released during tissue lesions [175]. TLR2 showed an upregulation in brain tissues of PD patients and involved in α-Syn-induced inflammatory responses, stimulating the release of pro-inflammatory cytokines [176, 177]. In TLR2 knockout (TLR2-/-) and wild-type (WT) mice treated with MPTP, He et al. found that deficiency of TLR2 significantly attenuated motor deficits and the nigrostriatal dopaminergic degeneration, and reduced astrocyte activation and neuroinflammation by inhibiting the TLR2/MyD88/TRAF6/NF-κB signaling pathway. Furthermore, TLR2 deficiency also increased the abundance of the protective genus *Prevotellaceae* and decreased the abundance of the genera *Oscillospira*, *Anaerotruncus*, *Lachnoclostridium*, and *Helicobacter*, which were associated with intestinal inflammation, implying that alterations of the gut microbiota in TLR2-deficient mice may contribute to the recovery of PD pathology [29]. TLR4, the bacterial endotoxin-specific ligand, is another TLR member that have been linked to the pathogenesis of PD. In the SN and medial temporal gyrus (GTM) of PD patients, TLR4 expression was upregulated and co-localized with phosphorylated α-Syn in DA neuronal LBs and Iba-1 in glial cells [178]. Perez-Pardo et al. found increased expression of TLR4, CD3^+^ T cells, cytokine in colonic biopsies and decreased abundance of SCFAs-producing bacteria in patients with PD. Intestinal inflammation, motor dysfunction, neuroinflammation, and neurodegeneration were less in rotenone-treated TLR4-KO mice compared to rotenone-treated WT mice [30]. However, deletion of TLR4 leaded to a ‘pro-inflammatory’ dysbiotic microbiota, including decreased relative abundance of the anti-inflammatory genera *Bifidobacterium* and/or *Lactobacillus* and increased relative abundance of the pro-inflammatory intestinal bacterial genera unclassified *Rickettsia*, *Coccidioides*, and *Lactobacillus*, which may be the reason that TLR4 deficiency could not completely protect against rotenone-induced PD pathology [30]. Further interventional studies, such as fecal transplantation or gene silencing of the TLR4 pathway, are required to establish a direct link between microbiota, TLR4, gut and CNS inflammation, and neurodegeneration in PD.

### Limitations

A large number of preclinical studies have demonstrated that some drugs and small molecules have protective effects on DA neuron damage in PD by affecting the composition and abundance of gut microbiota. We have summarized and discussed their roles in this review, but we have not conducted a direct and critical comparison between the different therapies discussed to determine which might be more promising or effective based on current evidence. In addition, further clarification of the drug’s half-life in the body, their abilities to cross the BBB, and dose toxicity is needed before it can be formally applied to clinical therapy.

## Conclusion and future directions

Although several studies have reported significant changes in the relative abundance of certain gut microbiota in the feces of patients with PD, the results often vary given differences in selection of patients and controls and the study methodology [179]. Future studies need to be greater harmonization and to use personalized approaches in the design of microbial-directed therapeutics. Numerous of preclinical studies have found that some small molecule drugs can exert neuroprotective and anti-inflammatory effects by restoring gut microbiota. However, the bioavailability of these small molecule compounds in in vivo and the effects on gut microbial metabolism and microbiota-gut-brain axis homeostasis are needed to be further explored. In addition, the duration and dose of treatment varied across studies, and more studies are needed to further refine the duration and dose of drug interventions.

Nowadays, macrogenomic microbial analyses matched with functional assays are crucial for the selection of therapeutic candidate molecules targeting PD pathology. Two mechanisms can be considered during the development of therapeutic strategies: (i) whether microbial release of molecules is able to reestablish gut and brain barrier homeostasis; and (ii) homeostasis of the brain-gut axis mediated by a remodeling of the host microbiota.

## Data Availability

Not applicable.
